# Health from grain: oat beta-glucan

**DOI:** 10.1080/16512235.2017.1343552

**Published:** 2017-07-03

**Authors:** Lovisa Martin Marais

**Affiliations:** ^a^ Lantmännen R&D, Stockholm, Sweden

Lantmännen is a Swedish cooperative owned by 27,000 farmers. Research has always been regarded as important within the cooperative; therefore, Lantmännen has its own research foundation allowing for investment in both external and internal research.

Oats are one food crop that has been studied because its components have possible health effects. It has been known since the nineteenth century that oats are good for you. The science behind beta-glucan in oats and its many health effects started to be unraveled in the late 1960s. Today, we know that the positive effects of beta-glucan include a number of important health parameters such as lowering cholesterol and lowering the blood sugar rise after a meal. At the same time, interest in food and prevention of diseases is growing among the general population.

Research is necessary for creating a platform for new possibilities and innovations. Beta-glucan is only one example of many unique molecules that research has uncovered. However, ‘we don’t eat molecules. We eat food’. To create increased health value in food production, a battery of knowledge from ‘field to fork’ is required.

The science of plant breeding is the very start of finding new varieties of cereal crops that can provide unique solutions in the future. For instance, oats are not just oats; every variety has its own name and characteristics. Some varieties have higher fat content, some higher protein content, and some have an increased amount of beta-glucan naturally occurring in the oat kernel. Plant breeding is an important step in producing consumer products that naturally contain more beta-glucan which, in turn, can offer the desired health effects after, for example, one bowl of oatmeal instead of two.

Farming of the crops is at the heart of the creation of cereal foods. The farmers’ knowledge over generations and constant development lead to improved quality, handling techniques, and standards for our cereal foods.

Product development teams discover technical, taste, and flavor solutions to meet consumer needs. With strong connections throughout the value chain, healthy food can reach the shelves, and if it is desirable and perceived as important enough, the health benefits will reach the consumer.

